# Human Physiology

**Published:** 1846-11

**Authors:** 


ART. VI.—*Human Physiology.* With three hundred and sixty-eight illustrations. By ROBLEY DUNGLISON, M.D., Professor of the Institutes of Medicine, in Jefferson Medical College, Philadelphia; Secretary to the American Philosophical Society, &c., &c. Sixth edition, greatly improved. In two volumes. 8vo. Philadelphia: Lea & Blanchard. 1846.

For several years the Physiology of Dr. Dunglison was almost without a rival in this country; within a few years the able works of Müller and Carpenter have been republished in Philadelphia, and have divided with it the favor which it enjoyed at its first appearing in an uncommon degree. But they have only shared in the public esteem, for we find that the American work has gone forward to its sixth edition. The author is not a man to suffer his books to fall behind the times; with his laborious industry they are sure to be brought to a level with the existing state of the science to which they refer. His Physiology is a work of unquestioned merit and ability, and is entitled to the rank which it has long enjoyed among the medical productions of the United States. It is written in a pleasing style, and abounds in knowledge interesting to every class of readers. It will continue to hold its place among the text-books for students during their attendance upon medical lectures.

